# Identifying County-Level All-Cause Mortality Rate Trajectories and Their Spatial Distribution Across the United States

**DOI:** 10.5888/pcd16.180486

**Published:** 2019-05-02

**Authors:** Peter Baltrus, Khusdeep Malhotra, George Rust, Robert Levine, Chaohua Li, Anne H. Gaglioti

**Affiliations:** 1 National Center for Primary Care, Morehouse School of Medicine, Atlanta, Georgia; 2 Department of Community Health and Preventive Medicine, Morehouse School of Medicine, Atlanta, Georgia; 3 Geography and Urban Studies, Temple University, Philadelphia, Pennsylvania; 4 Department of Behavioral Sciences and Social Medicine, Florida State University School of Medicine, Tallahassee, Florida; 5 Department of Family and Community Medicine, Baylor College of Medicine, Houston, Texas; 6 Department of Family Medicine, Morehouse School of Medicine, Atlanta, Georgia

## Abstract

**Introduction:**

All-cause mortality in the United States declined from 1935 through 2014, with a recent uptick in 2015. This national trend is composed of disparate local trends. We identified distinct groups of all-cause mortality rate trajectories by grouping US counties with similar temporal trajectories.

**Methods:**

We used all-cause mortality rates in all US counties for 1999 through 2016 and estimated discrete mixture models by using county level mortality rates. Proc Traj in SAS was used to detect how county trajectories clustered into groups on the basis of similar intercepts, slopes, and higher order terms. Models with increasing numbers of groups were assessed on the basis of model fit. We created county-level maps of mortality trajectory groups by using ArcGIS.

**Results:**

Eight unique trajectory groups were detected among 3,091 counties. The average mortality rate in the most favorable trajectory group declined 29.4%, from 592.3 deaths per 100,000 in 1999 to 418.2 in 2016. The least favorable mortality trajectory group declined 3.4% over the period, from 1,280.3 deaths per 100,000 to 1,236.9. We saw significant differences in the demographic and socioeconomic profiles and geographic patterns across the trajectory categories, with favorable mortality trajectories in the Northeast, Midwest, and on the West Coast and unfavorable trajectories concentrated in the Southeast.

**Conclusions:**

County-level disparities in all-cause mortality rates widened over the past 18 years. Further investigation of the determinants of the trajectory groupings and the geographic outliers identified by our research could inform interventions to achieve equitable distribution of county mortality rates.

**Summary**

**What is already known about this topic?**

All-cause mortality in the United States declined from 1935 through 2014, with a recent uptick in 2015. This national trend is composed of disparate local trends.

**What is added by this report?**

By using a novel methodology, we detected 8 unique county-level mortality rate trajectory groups. Disparities widened from 1999 to 2016. Differences existed in the demographic and socioeconomic profiles across the trajectory groups, with favorable mortality trajectories in the Northeast, in the Midwest, and on the West Coast and unfavorable trajectories concentrated in the Southeast.

**What are the implications for public health practice?**

Further investigation of the determinants of the trajectory groupings and the geographic outliers identified could inform interventions to achieve equitable distribution of county mortality rates.

## Introduction

The all-cause mortality rate is an indicator of general population health. The age-adjusted all-cause mortality rate declined in the US general population from 1935 (1) to a record low in 2014 (2). A notable 1.1% increase occurred in the age-adjusted all-cause mortality rate in 2015 (3). Overall declines in mortality rates did not occur in all geographic areas (4); southeastern states had higher rates overall and lower rates of decline compared with the national trend (3).

Although mortality rate trends differ by state, it is important to study mortality and mortality trends at smaller geographic levels. Although use of counties as a geographic unit of analysis has limitations (5,6) and county-level infrastructure is variable, counties are the smallest unit of analysis for which stable mortality rates can be calculated and for which infrastructure exists for implementing and administering health and social policies. County mortality rates vary by geography (7,8), but few analyses of all-cause mortality rate trends have been done at the county level. Although some methods are available to compare and analyze long-term trends in mortality rates that include joinpoint regression, spatial and aspatial generalized linear mixed models, and Bayesian space–time models, all these approaches rely on the change in the rates being compared to exhibit linear or log linear changes over time and rely on a series of changes between small intervals over the entire time period (9).

We sought to group and examine common trends in county-level mortality for the most recently available mortality data (1999–2016) by using a new statistical method called group-based trajectory modeling (GBTM). Although trends in US counties were previously reported by examining the difference in rates at 2 time points and linear or log linear changes in rates over time, GBTM incorporates information from all time points and allows for examination of nonlinear (quadratic, cubic, and other higher order) rate trends. GBTM determines if groups of study units with similar trajectory shapes exist and has been used to determine whether the health outcome trends of individual units group together into patterns (10–13). To our knowledge, this method has not been used to examine mortality rates in US counties.

We sought to identify patterns of county mortality rate trajectories and to determine if any positive (exceptionally low initial rates decreasing rapidly) or negative (exceptionally high rates decreasing slowly or not at all) deviant trajectories existed. We also estimated the extent to which trajectories clustered geographically. Finally, we identified geographic deviants: counties whose mortality rate trajectory group patterns were significantly different than the trajectories of surrounding counties.

## Methods

County-level, age adjusted mortality data from the Compressed Mortality File was obtained for years 1999 through 2016 from the National Center for Health Statistics through a data use agreement (14). We included all deaths across the entire age spectrum. We included rates for each year in which the number of deaths in a county was greater than or equal to 20. Counties were included in the analysis if they had at least 2 years of stable mortality rate data.

The yearly, age-adjusted, all-cause mortality rate of the county was the outcome measure used to generate rate trajectories using Proc Traj for SAS, version 9.4 (SAS Institute, Inc) (15,16). Group-based trajectory modeling assumes that a certain number of discrete underlying groups in the population each have their own population prevalence, intercept, and slope and possibly higher order terms (17). These subpopulations are not directly observable but are estimated (latent).

**Figure 1:**
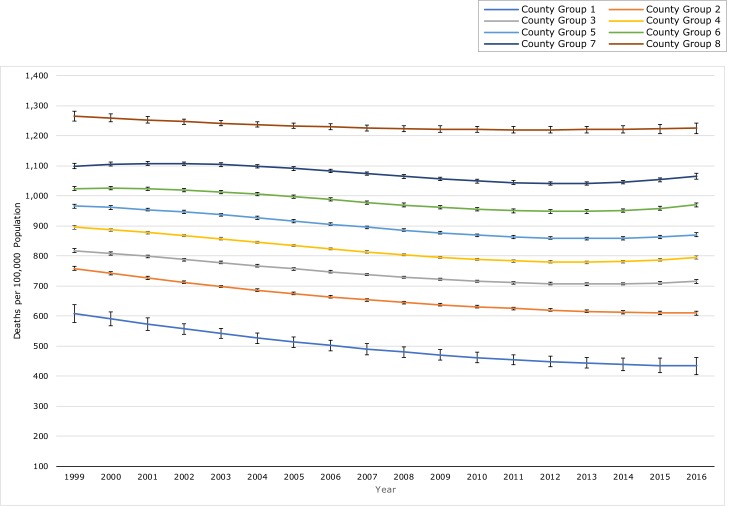
Age-adjusted mortality rate trajectories for US counties for 8 groups of counties based on group-based trajectory models, 1999–2016. The outcome measure used to generate rate trajectories was the yearly, age-adjusted, all-cause mortality rate of the county. Solid lines correspond to model-predicted values for rates; dotted lines are confidence intervals for the predicted values.

Proc Traj requires specification of the number of groups the model will fit. We estimated a quadratic model with a dependent variable of mortality rate and an independent variable of time in years with a single group and kept adding groups and assessing the change in the Bayesian information criterion (BIC) as an evaluation of model fit (15,18). We simultaneously assessed the percentage of counties in each group and the shape of the trajectories when plotted. The fit of the model increased with the addition of more groups. The model with 8 groups produced both a negative deviant group and a positive deviant group (defined as being less than 2% of the counties and substantially different upon visual inspection from the other trajectories). Group 1 was the positive deviant group whereas group 8 was the negative deviant group, both having trajectories with substantially lower rates (group 1) or higher rates (group 8) than the rest of the trajectories (Figure 1). Identification of such groups was one of the aims of our study; adding a greater number of groups did not affect the composition of these 2 groups, nor did it identify any new deviant groups. Including more than 8 groups only created more roughly parallel groups between group 2 and 7, some with very small numbers of counties. The BIC continued to increase with the addition of more groups beyond 8 (Appendix A), but on the basis of the foregoing considerations we stopped at 8 groups for ease of interpretation of the data. For sensitivity analysis, we repeated the process with linear models as the starting point. Trajectory groups looked similar to linear models, but the quadratic models produced a better fit according to the BIC. We next added or removed second and higher-order terms from each group’s model on the basis of significance (*P* < .05). This process yielded quadratic models for trajectories 1, 2, and 8. Trajectories 3 through 7 included a cubic term.

**Table:** 

Year	Group 1	Group 2	Group 3	Group 4	Group 5	Group 6	Group 7	Group 8
**1999**	609.1 (579.8–638.4)	758.6 (751.9–765.4)	818.1 (811.9–824.4)	895.2 (889.5–900.9)	966.0 (959.3–972.7)	1,024.7 (1,017.5–1,031.9)	1,098.9 (1,089.6–1,108.1)	1,266.1 (1,249.8–1,282.3)
**2000**	590.7 (566.6–614.8)	742.3 (736.74–747.8)	808.7 (804.0–813.5)	887.3 (883.0–891.7)	961.1 (955.6–966.7)	1,025.6 (1,020.0–1,031.2)	1,105.4 (1,098.5–1,112.4)	1,259.2 (1,246.1–1,272.2)
**2001**	573.3 (552.9–593.7)	726.9 (722.2–731.6)	798.8 (794.5–803.0)	878.2 (874.3–882.1)	954.4 (949.1–959.7)	1,023.6 (1018.5–1028.8)	1,108.09 (1102.1–1114.1)	1,252.8 (1242.1–1263.5)
**2002**	556.9 (538.8–575.1)	712.4 (708.2–716.6)	788.4 (784.2–792.7)	868.05 (864.1–872.00)	946.2 (940.9–951.5)	1,019.3 (1014.1–1024.6)	1,107.36 (1101.4–1113.3)	1,247.0 (1237.8–1256.3)
**2003**	541.6 (524.3–558.8)	698.9 (694.9–702.9)	777.9 (773.7–782.2)	857.2 (853.2–861.3)	936.9 (931.6–942.3)	1013.07 (1,007.6–1,018.5)	1,103.83 (1,097.8–1,109.9)	1,241.8 (1,233.1–1,250.5)
**2004**	527.2 (510.0–544.5)	686.4 (682.4–690.4)	767.5 (763.2–771.8)	846.0 (842.0–850.1)	926.8 (921.6–932.1)	1,005.4 (999.9–1011.0)	1,098.08 (1,092.0–1,104.2)	1,237.2 (1,228.3–1246.0)
**2005**	513.9 (496.2–531.6)	674.8 (670.7–678.9)	757.2 (753.1–761.4)	834.8 (830.8–838.8)	916.3 (911.2–921.4)	996.8 (991.2–1,002.3)	1,090.69 (1,084.8–1,096.6)	1,23315 (1,223.8–1,242.3)
**2006**	501.6 (483.4–519.7)	664.1 (659.9–668.4)	747.4 (743.4–751.4)	823.8 (819.8–827.7)	905.7 (900.9–910.6)	987.6 (982.1–993.2)	1,082.23 (1,076.5–1,088.0)	1,229.5 (1,219.8–1,239.2)
**2007**	490.2 (471.8–508.7)	654.4 (650.05–658.8)	738.2 (734.3–742.1)	813.3 (809.4–817.2)	895.4 (890.7–900.0)	978.4 (972.8–984.0)	1,073.3 (1,067.7–1,078.9)	1,226.5 (1,216.47–1,236.56)
**2008**	479.9 (461.5–498.4)	645.6 (641.3–650.0)	729.8 (725.9–733.7)	803.7 (799.8–807.6)	885.6 (881.17–890.2)	969.6 (963.9–975.3)	1,064.4 (1,058.8–1,070.1)	1,224.1 (1,213.9–1,234.3)
**2009**	470.6 (452.5–488.8)	637.8 (633.5–642.2)	722.4 (718.5–726.4)	795.3 (791.3–799.3)	876.8 (872.3–881.4)	961.7 (955.8–967.6)	1,056.2 (1,050.43–1,062.01)	1,222.2 (1,212.0–1,232.5)
**2010**	462.4 (444.7–480.0)	631.0 (626.7–635.3)	716.2 (712.2–720.3)	788.4 (784.3–792.6)	869.3 (864.7–873.9)	955.2 (949.1–961.3)	1,049.3 (1,043.3–1,055.3)	1,220.9 (1,210.9–1,231.1)
**2011**	455.1 (437.8–472.3)	625.1 (620.8–629.3)	711.45 (707.3–715.6)	783.3 (779.0–787.6)	863.43 (858.7–868.2)	950.4 (944.2–956.6)	1,044.1 (1,038.0.0–1,050.3)	1,220.1 (1,209.9–1,230.3)
**2012**	448.8 (431.6–466.0)	620.07 (615.73–624.41)	708.26 (704.09–712.44)	780.30 (775.99–784.61)	859.54 (854.69–864.39)	947.91 (941.73–954.08)	1041.39 (1035.21–1047.57)	1219.93 (1209.44–1230.41)
**2013**	443.56 (425.44–461.69)	616.1 (611.5–620.7)	706.9 (702.7–711.0)	779.7 (775.4–784.0)	858.0 (853.0–862.9)	948.2 (942.1–954.2)	1,041.6 (1,035.5–1,047.7)	1,220.3 (1,209.0–1,231.5)
**2014**	439.3 (419.0–459.7)	613.0 (607.8–618.1)	707.5 (703.3–711.7)	781.9 (777.5–786.3)	859.1 (853.9–864.3)	951.6 (945.7–957.5)	1,045.4 (1039.1–1051.7)	1,221.2 (1,208.5–1,233.9)
**2015**	436.1 (412.0–460.2)	610.9 (604.9–616.9)	710.3 (705.5–715.0)	787.1 (782.3–791.9)	863.2 (857.3–869.1)	958.7 (952.6–964.8)	1,053.31 (1,045.9–1,060.7)	1,222.7 (1,207.7–1,237.77)
**2016**	433.88 (404.64–463.12)	609.69 (602.53–616.85)	715.44 (709.23–721.65)	795.70 (789.68–801.71)	870.72 (863.32–878.13)	969.93 (962.45–977.41)	1065.92 (1055.95–1075.90)	1224.7 (1206.67–1242.73)

We used US census data for 2000 and 2010 to describe the changes in sociodemographic composition of the county trajectory groups. Variables included total population, population density (population per square mile), median age, percentage of county population living below the federal poverty level, median household income, percentage white population, percentage black population, percentage American Indian/Alaska Native population, percentage Asian population, and percentage Hispanic (any race) population. We reported means for each year and changes of means between the years.

**Figure 2:**
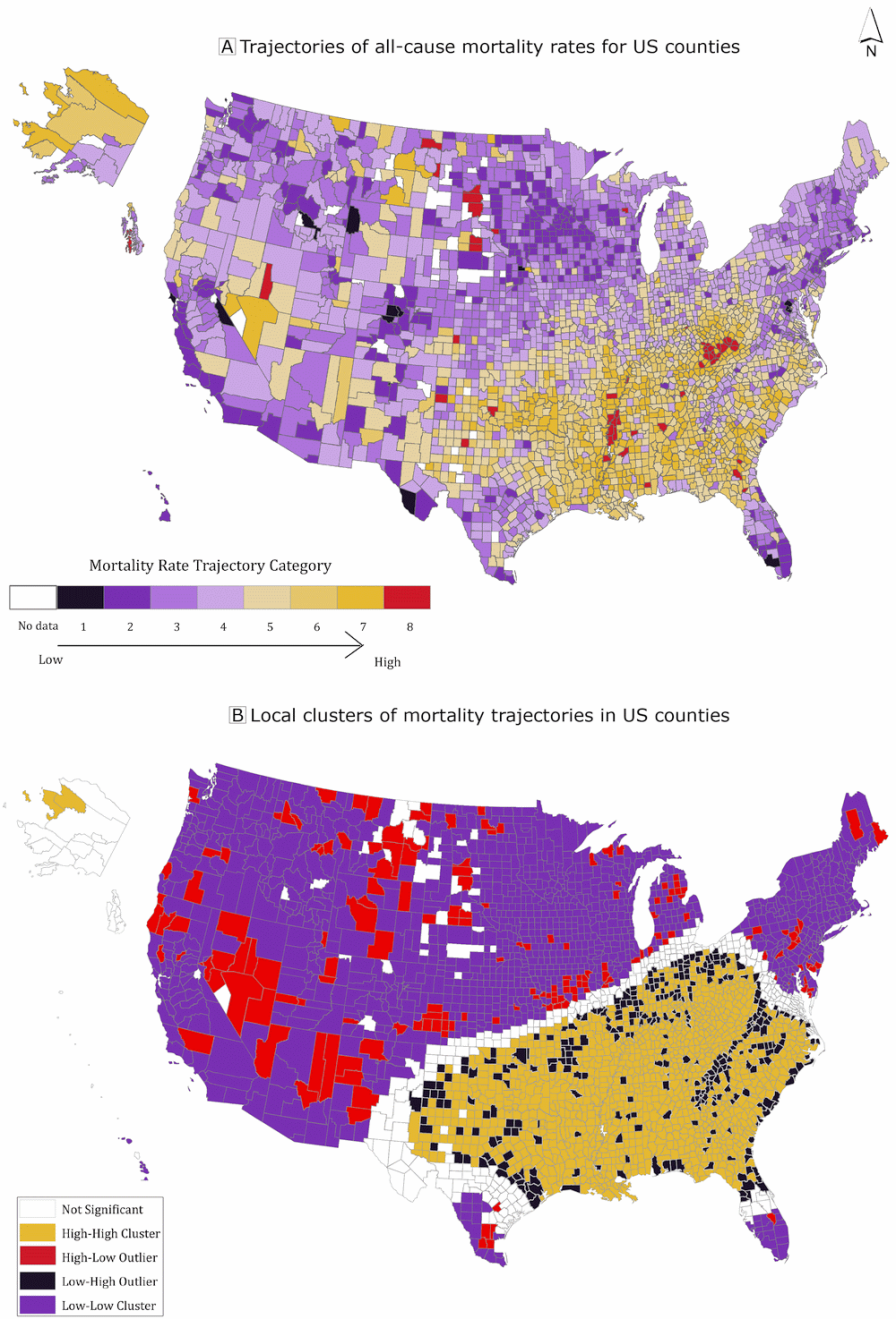
Trajectories of age-adjusted all-cause mortality in US counties using group-based trajectory models, 1999–2016. The outcome measure used to generate rate trajectories was the yearly, age-adjusted, all-cause mortality rate of the county. Panel A: Trajectories of all-cause mortality rates for US counties. Panel B: Local clusters of mortality trajectories in US counties detected by using local indicators of spatial association (LISA). The 4 categories of significant spatial clusters (*P* < .05): 1) high–high clusters representing all counties with high mortality, the worst trajectory group; 2) high–low outliers representing counties in the worst trajectory groups near counties in the most favorable trajectory groups (at-risk counties doing worse than those around them); 3) low–high outliers representing counties in the best trajectory groups near counties in the worst trajectory groups (resilient counties doing better than those around them); and 4) low–low clusters of counties in the most favorable trajectory groups. Source: 1999–2016 Compressed Mortality File, Centers for Disease Control and Prevention (14).

We created a choropleth map of the county trajectory groups (Figure 2). Thematic mapping of county trajectories showed clear evidence of spatial autocorrelation. This simply means that observations that are located next to each other are related to each other, that is, there is no spatial independence between observations. We measured the degree of spatial autocorrelation (ie, the degree to which neighboring observations are related to each other) by using the Global Moran’s I statistic of ArcGIS Pro (Esri). We used 2 method to determine the number of neighbors for each observation: polygon contiguity (based on neighbors sharing borders) and inverse distance (which means the farther away a neighbor is, the less influence the neighbor has) (19,20). Once we determined the number and relationship of neighbors, we identified local clusters by using the local indicators of spatial association (LISA) technique (19). The LISA technique generates a statistic named Getis-Ord Gi* (Esri), which specifies where features with high or low values cluster. Significant clusters were those where a feature and its neighbors all had high Getis-Ord Gi* values. Geographic deviants were defined as counties that had much higher or much lower values than their neighboring counties. On the basis of a county’s relative position within a cluster, counties were grouped into 4 categories of significant spatial clusters (*P* < .05): 1) high–high clusters representing all counties with high mortality, the worst trajectory group; 2) high–low clusters representing counties in the worst trajectory groups near counties in the most favorable trajectory groups (at-risk counties doing worse than those around them); 3) low–high clusters representing counties in the best trajectory groups near counties in the worst trajectory groups (resilient counties doing better than those around them); and 4) low–low clusters of counties in the most favorable trajectory groups. Of 3,144 counties, 3,091 counties and county equivalents were included in the analysis.

## Results

The equations for trajectories 1, 2, and 8 included quadratic terms, which produced trajectories with slower mortality rates decline over time (Figure 1). The equations for trajectories 3 through 7 contained a cubic term and produced trajectories that had a slowing rate of decline in rates with increasing rates near the end of the study period (Table 1). The numeric ordering of trajectories reflects mortality rate trajectories from most favorable to least favorable. Trajectory 1 had the lowest average mortality rate at the beginning of the study (1999) and at the end of the study (2016) and the steepest decline over the study period. Trajectory 8 had the highest mortality rates at both time points and only a modest decline over the study period. The trajectories did not overlap, which indicates that disparities in mortality rates across the trajectory groups persisted throughout the study period.

**Table 1:** Coefficients for Estimated Trajectories From Group-Based Trajectory Models Using 1999–2016 US County Annual All-Cause Mortality Data^a^

Trajectory^b^	Intercept^c^ (*P* Value)	Slope^d^ (*P* Value)	Quadratic^e^ (*P* Value)	Cubic^f^ (*P* Value)	% of US Counties	No. of Counties
1	628.51 (<.001)	−19.92 (<.001)	0.51 (.02)	NA	0.5	14
2	775.94 (<.001)	−17.78 (<.001)	0.47 (<.001)	NA	9.4	290
3	826.74 (<.001)	−8.19 (<.001)	−0.48 (.03)	0.03 (<.001)	19.7	608
4	901.42 (<.001)	−5.31 (.002)	−0.96 (<.001)	0.05 (<.001)	25.2	780
5	968.73 (<.001)	−1.53 (.42)	−1.25 (<.001)	.05 (<.001)	20.3	626
6	1,020.59 (<.001)	5.95 (.007)	−1.88 (<.001)	0.08 (<.001)	15.1	467
7	1,087.72 (<.001)	13.58 (<.001)	−2.55 (<.001)	0.10 (<.001)	8.2	252
8	1,273.51 (<.001)	−7.73 (.002)	0.28 (.03)	NA	1.7	54

Abbreviation: NA, not applicable.

a Coefficients are from an 8-group model; coefficients were added or removed from models if *P* < .05 for the coefficient. Note that if a term became nonsignificant when a higher-order term was added to the model and significant, the nonsignificant lower-order term remained in the model. Data are from the Centers for Disease Control and Prevention’s Compressed Mortality File (14).

b The numeric ordering of trajectories reflects mortality rates from most favorable (lowest baseline rate/largest decline in rate) to least favorable (highest baseline rate/largest decline in rate).

c Baseline mortality rate estimated by the model.

d First order term estimated by model; represents the linear component of change in rate per year.

e Second order term estimated by model; represents the quadratic component of change in rate per year.

f Third order term estimated by model (if significant); represents the cubic component of change in rate per year.

Disparities between trajectory groups increased over the study period. At baseline, the average mortality rate for Trajectory 1 was 592.3 deaths per 100,000, decreasing by 29.4% to 418.2 deaths per 100,000, whereas Trajectory 8 had a baseline rate of 1,280.3 deaths per 100,000 and decreased by 3.4% over the 18-year period to 1,236.9 deaths per 100,000 (Table 2). These 2 groups had a difference of 688 deaths per 100,000 in 1999 that increased to a difference of 818.7 deaths per 100,000 in 2016. There was a graded association in the amount of change in rates across the trajectory groups; as baseline rates increased, the rate decline decreased.

**Table 2:** Age-Adjusted, All-Cause Mortality Rates and Demographic Characteristics of the 1999–2016 County Mortality Trajectory Groups^a^

County Group	1	2	3	4	5	6	7	8	Overall
**Counties, n (%)**	14 (0.5)	290 (9.4)	608 (19.7)	780 (25.2)	626 (20.3)	467 (15.1)	252 (8.2)	54 (1.7)	3091 (100)
**Mortality rate per 100,000**									
1999	592.3	748.6	813.4	893.2	961.6	1,026.7	1,104.9	1,280.3	920.1
2016	418.2	604.7	709.5	789.1	863.3	964.3	1,059.9	1,236.9	825.2
Change	−173.9	−143.9	−103.9	−104.1	−98.3	−62.4	−45.0	−43.4	−94.9
% Change	−29.4	−19.2	−12.8	−11.7	−10.2	−6.1	−4.1	−3.4	−10.3
**Population**									
2000	180,108	231,865	106,542	99,164	62,933	46,473	30,796	20,059	91,196
2010	202,341	255,498	120,595	109,220	68,043	47,651	30,859	18,939	100,053
Change	22,234	24,324	13,888	10,087	5,111	1,088	633	−857	8,853
**Population density^b^**									
2000	370.3	839.7	224.3	212.8	167.7	144.1	146.0	68.1	247.3
2010	413.0	903.5	247.3	227.8	181.2	143.8	143.0	64.5	264.2
Change	42.7	63.8	22.6	15.0	13.5	−0.3	−3.1	−2.4	17.2
**Median age, y**									
2000	36.8	37.4	37.9	37.3	37.4	36.9	36.4	33.4	37.2
2010	39.9	40.6	41.1	40.4	40.1	39.5	39.3	37.3	40.2
Change	3.1	3.2	3.2	3.0	2.7	2.6	2.9	3.8	2.9
**Persons with income below federal poverty level, %**									
2000	8.0	9.0	10.1	11.9	14.1	16.6	19.6	23.9	13.3
2010	10.5	11.7	13.1	15.3	17.8	20.5	24.4	28.8	16.8
Change	2.4	2.8	3.0	3.3	3.7	3.9	4.8	4.9	3.5
**Median annual household income^c^, $ **									
2000	83,248	66,912	59,120	55,160	50,636	45,790	41,360	37,317	53,400
2010	76,184	62,960	55,007	50,575	46,311	42,078	37,987	36,653	49,308
Change	−7,064	−3,952	−4,113	−4,585	−4,325	−3,712	−3,373	−664	−4,092
**Race/ethnicity, %**									
**White**									
2000	86.6	88.2	91.1	87.9	83.4	77.8	71.2	70.1	84.4
2010	83.4	86.1	89.5	86.6	82.0	76.4	69.4	68.4	82.9
Change	−3.2	−2.1	−1.7	−1.3	−1.4	−1.4	−1.7	−1.4	−1.5
**Black**									
2000	2.5	2.9	2.4	5.2	10.9	16.5	22.3	16.3	8.9
2010	3.0	3.2	2.8	5.4	10.9	16.3	22.5	16.2	9.0
Change	0.4	0.3	0.4	0.2	−0.1	−0.2	0.2	−0.2	0.1
**Asian**									
2000	2.8	2.6	1.0	0.7	0.5	0.4	0.3	0.3	0.8
2010	3.8	3.4	1.4	1.0	0.7	0.6	0.4	0.3	1.2
Change	1.0	0.8	0.4	0.3	0.2	0.2	0.1	0.1	0.3
**American Indian/Alaska Native**									
2000	0.6	0.8	1.2	1.4	1.2	2.2	3.9	11.8	1.8
2010	0.6	0.9	1.3	1.5	1.3	2.4	4.1	12.7	1.9
Change	0.1	0.1	0.1	0.1	0.1	0.2	0.2	0.4	0.1
**Hispanic^d^**									
2000	16.3	8.3	7.0	7.3	6.2	3.8	2.3	2.00	6.1
2010	21.2	10.8	9.3	9.5	8.5	5.8	3.5	2.7	8.3
Change	4.9	2.5	2.3	2.2	2.3	2.0	1.2	0.7	2.1

a Data are from the Centers for Disease Control and Prevention’s Compressed Mortality File (14). Sociodemographic data are from the 2000 and 2010 US Census.

b People per square mile.

c 2018 dollars.

d Any race.

Sociodemographic characteristics of county trajectory groupings were similar for 2000 and 2010. A graded association with median income and poverty was noted across trajectory groups. Median income decreased and percentage of county population living below the federal poverty level increased as health trajectories worsened (Table 2). A more complex relationship was observed with racial composition of mortality trajectory groupings. The county percentage of black population increased from trajectory 1 to trajectory 7. Percentage of white population increased across Trajectories 1 to 2, peaked at Trajectory 3, and then decreased from Trajectory 3 to 8. The percentage of American Indian/Alaska Native population increased across trajectory groups, peaking in Trajectory 8 (2000,11.8%; 2010,12.7%). The percentage of Asian and Hispanic populations in county trajectory groups increased as trajectories became more favorable.

Panel A of Figure 2 depicts the geographic variation of mortality rate trajectory groups. The Southeast was characterized by counties in high mortality rate trajectory groups, whereas counties in low mortality trajectory groups tended to be in the Northeast, the upper Midwest, and the West Coast. This pattern was reflected in the clustering of counties detected by using LISA (Panel B of Figure 2). Clusters of the most favorable trajectory counties (low–low) and counties with worse trajectories than neighboring counties (high–low) were in the northern, midwestern, and western regions. Clusters of the least favorable trajectory counties (high–high) and clusters of counties with significantly better trajectories than their neighboring counties (low–high) were predominantly in the south. This indicates that while some regions of the country may be doing well or poorly in terms of mortality there are counties with substantially different mortality trajectory patterns than their geographic neighbors.

We identified positive and negative deviant county groups. Trajectory 1 (positive deviant, n = 14) had substantially lower mortality rates than the middle 6 trajectories, and trajectory 8 (negative deviant, n = 50) had substantially higher mortality rates than the middle 6 trajectories during the study period. Positive deviant counties tended to be wealthy except for Presidio County, Texas, a small, West Texas county bordering the Rio Grande River with a largely Hispanic (83.4%) population. Two trajectory 1 counties were in the Washington, District of Columbia, metropolitan area and 3 in Colorado; the remaining trajectory 1 counties were dispersed throughout the country. Several negative deviant counties were identified with differing demographic characteristics, but most had high poverty rates. Counties in North Dakota (n = 1) and South Dakota (n = 5) had large Native American populations, counties in the Mississippi Delta (n = 8) had large black populations, and an Appalachian cluster in Kentucky (n = 14) and West Virginia (n = 4) was predominantly white.

## Discussion

We used a new application of group-based trajectory modeling to identify groups of US counties with similar temporal trajectories of all-cause mortality rates. This national analysis over an 18-year period identified 8 distinct trajectory groups. Within those trajectories, we identified groups of positive and negative deviant counties. This work presents a new approach to identifying and quantifying spatiotemporal trends in health disparities that addresses limitations of current approaches. First, this approach overcomes the limitation of relying on linear or log-linear rate changes over time by allowing for higher order terms in the equations used to generate trajectories. Second, this approach allows the use of all rates in a period instead of relying on change between rates at 2 points within an overall period. Third, this approach groups trajectory patterns that emerge from the data used to support the analysis instead of relying on an a priori trend categorization. Our main findings show substantial and widening inequities in mortality rates and mortality rate trends across groups of US counties. We saw geographic clustering of the trajectories, with worse trajectories clustering in the Southeast and better trajectories clustering in the Northeast, the upper Midwest, and the West Coast. Local-area variation in mortality has been well-documented in the United States (7,8). However, identification of clusters of counties with similar mortality rate trajectories over time contributes to understanding the factors that drive such differences. Demographic factors such as racial composition and socioeconomic status have been demonstrated and partially explain high mortality trajectories and less favorable mortality trajectories in the South (21).

In our analysis, several counties in trajectory 8, the worst mortality trajectory group, have disproportionately large American Indian populations. For example, Sioux County, North Dakota, rests entirely within the Standing Rock Indian Reservation. Buffalo County, South Dakota, where the Cow Creek Sioux Tribe resides, had the highest 2016 all-cause mortality rate and the lowest per capita income in the United States. This may be because American Indians have higher rates of mortality across the lifespan than other racial/ethnic groups (22–24). Additionally, the economic and social conditions on reservations may contribute to a higher mortality rate and a less favorable temporal mortality rate trajectory for American Indians living on reservations compared with those living in other areas of the country.

Historically disenfranchised places in the Mississippi Delta, where there were high concentrations of slavery followed by the structural inequities of sharecropping and segregation (25), and in Appalachia, where poverty and environmental and occupational injustice is entrenched (26), had a disproportionate number of trajectory 8 counties. One study found similar spatial clustering of poor physical and mental health and food insecurity in these areas (27). Counties in trajectory 8 that were not part of geographic clusters may have unique factors that explain their poor mortality rates and rate trajectories that warrant further exploration. The size of the rate gap between trajectory 8 counties and the other trajectory groupings is cause for concern, further study, and action.

Counties in the best trajectory group, trajectory 1, had generally higher socioeconomic conditions than other parts of the country, but not uniformly so. Marin County, California; Los Alamos, New Mexico; Montgomery County, Maryland; and Fairfax County, Virginia, ranked in the top 20 counties in the nation by median income. No other county in the top 25 median-income counties for the nation was found in this best outcome group, so high socioeconomic status may not be enough to predict favorable mortality trajectory trends. Other counties in the group had a less affluent socioeconomic profile. For example, although Collier County, Florida, includes affluent communities such as Naples and Marco Island, it also included vast rural areas with large numbers of migrant farmworkers and had an overall median income less than half that of the most affluent counties in the nation.

Multilevel influences potentially contribute to the differences we observed across groups of mortality rate trajectories. Changes in socioeconomic status, demographic composition, health care infrastructure, patterns of health care use, health behaviors, and changes in state and federal health, housing, education, and social policy could all be contributing factors. One demographic compositional change we noted was that the largest percentage and change in percentage of Hispanic populations occurred in counties with the best mortality outcomes. This may be due to the documented “Hispanic paradox” in health outcomes (28,29).

Although we saw a significant geographic clustering of counties in each trajectory, some counties with low mortality rate trajectories were in the same geographic area as those with high rate trajectories (and vice versa). These counties may be considered positive deviants, having achieved more optimal mortality rates and rate trends despite being surrounded by counties with worse mortality rates and less improvement over time. If these positive deviance communities have common characteristics amenable to intervention, they could reveal a path toward achieving improved outcomes in counties with unfavorable trajectory patterns. Alternatively, these positive deviant counties may be surrounded by counties with significantly different demographic composition, health care access, or rurality, and such differences also may account for the differences in mortality trajectories observed in our analysis.

Our study has several limitations. We chose to use age-adjusted mortality rates for everyone without stratifying by age, sex, or race to create an overall indicator of public health in US counties because of the large amount of space required to present a description of this novel methodology for the first time. Preliminary analysis of different age and racial/ethnic groups has indeed revealed nonuniform trends (Appendix B), which we intend to discuss in future articles. By studying all-cause mortality, differences in specific causes of death would possibly cause different trajectory groupings and geographic patterns. On the other hand, all-cause mortality is less subject to many of the known limitations of death certificate data. We have only begun to tease out the myriad explanatory factors for these differences in outcomes. Although geographic granularity is limited in this county-level analysis, smaller neighborhood-level analyses may produce unstable rates and may be difficult to interpret on a national level. There are also limitations in interpreting the results of the statistical models. Trajectory 1 contained only 14 counties, but these counties had a greater than 98% probability of belonging to group 1, indicating that they are true outliers. All counties had a greater than 50% probability of membership in their assigned group, and misclassification would likely result in being assigned to the trajectory above or below the one reported. More groups could have been added to the model, but this would have improved the model fit minimally without providing more information to inform interventions.

Further research should examine what county level factors are associated with the observed patterns in county groupings of mortality rate trajectories identified here. Demographic, socioeconomic, and health system variables as well as social variables such as social capital and social cohesion should be examined. Although traditional regression models will be helpful, we suggest that a more comprehensive approach be taken to determine how these variables interact to produce the observed patterns. Such an approach will require the use of longitudinal data on the predictor variables and modeling approaches including multilevel modeling, structural equations, and system dynamic models.

That county disparities in temporal, all-cause mortality rate trends are worsening suggests that we need to quickly learn the reasons why some counties succeed in reducing mortality rates while others fail. The lessons learned from successful counties could be applied to those that are failing. The identification of positive geographic outliers may provide an opportunity to learn what factors may be driving exceptional outcomes. Hopefully, investigating these special cases will lead to knowledge to help improve the health outcomes of lagging counties and thereby reduce county level disparities in the all-cause mortality trends observed here.

## Appendix A Bayesian Information Criterion For Models With 1 to 30 Groups of Counties^a^

**Table:**

Number of Group	BIC	∆BIC^b^
1	−353,621.1	Not applicable
2	−33,8347.9	15,273.2
3	−33,2614.9	5,733
4	−32,9808.4	2,806.5
5	−32,8178.7	1,629.7
6	−32,7229.1	949.6
7	−32,6665.9	563.2
8	−32,6316.9	349.0
9	−32,5754.4	562.5
10	−32,5551.7	202.7
11	−32,5567.8	−16.1
12	−32,5230.3	337.5
13	−32,5012.4	217.9
14	−32,4841.7	170.7
15	−32,4743.4	98.3
16	−32,4601.8	141.6
17	−32,4544.4	57.4
18	−32,4481.9	62.5
19	−32,4438.9	43.0
20	−32,4460.2	−21.3
21	−32,4318.0	142.2
22	−32,4311.6	6.4
23	−32,4204.7	106.9
24	−32,4214.5	−9.8
25	−32,4179.4	35.1
26	−32,4121.1	58.3
27	−32,4084.9	36.2
28	−32,4188.9	−104.0
29	−32,4123.2	65.7
30	−32,4152.1	−28.9

^a^ All models are quadratic.

^b^ ∆BIC = BIC_group = k+1 _– BIC_group = k_.

## Appendix B Trajectories for County All-Cause Mortality Rates, 1999–2016, by Race/Ethnicity, Age Groups, and Sex.

This appendix is available for download at https://www.msm.edu/Research/research_centersandinstitutes/NCPC2/documents/publications/Preventing-Chronic-Disease-Appendix-B.pdf

## References

[1] Hoyert D . 75 years of mortality in the United States, 1935–2010. NCHS Data Brief*.* Vol 88. Hyattsville (MD): National Center for Health Statistics; 2012. 22617094

[2] Kochanek KD , Murphy SL , Xu J , Tejada-Vera B . Deaths: final data for 2014. Natl Vital Stat Rep 2016;65(4):1–122. 27378572

[3] Murphy SL , Xu J , Kochanek KD , Curtin SC , Arias E . Deaths: final data for 2015. 2017. https://stacks.cdc.gov/view/cdc/50011. Accessed March 22, 2019. 29235985

[4] Levine R , Briggs N , Husaini B , Hennekens C . Geographic studies of black–white mortality. In: Satcher D, Pamies R, editors. Multicultural medicine and health disparities. New York (NY): MacMillan and Co; 2006.

[5] Krieger N , Chen JT , Waterman PD , Soobader M-J , Subramanian SV , Carson R . Geocoding and monitoring of US socioeconomic inequalities in mortality and cancer incidence: does the choice of area-based measure and geographic level matter?: the Public Health Disparities Geocoding Project. Am J Epidemiol 2002;156(5):471–82. 10.1093/aje/kwf068 12196317

[6] Chen JT , Rehkopf DH , Waterman PD , Subramanian SV , Coull BA , Cohen B , Mapping and measuring social disparities in premature mortality: the impact of census tract poverty within and across Boston neighborhoods, 1999–2001. J Urban Health 2006;83(6):1063–84. 10.1007/s11524-006-9089-7 17001522 PMC3261292

[7] Murray CJ , Kulkarni SC , Michaud C , Tomijima N , Bulzacchelli MT , Iandiorio TJ , Eight Americas: investigating mortality disparities across races, counties, and race-counties in the United States. PLoS Med 2006;3(9):e260. Erratum in: PLoS Med 2006;3(12):e545. . 10.1371/journal.pmed.0030260 16968116 PMC1564165

[8] Cullen MR , Cummins C , Fuchs VR . Geographic and racial variation in premature mortality in the U.S.: analyzing the disparities. PLoS One 2012;7(4):e32930. 10.1371/journal.pone.0032930 22529892 PMC3328498

[9] Vaughan AS , Kramer MR , Waller LA , Schieb LJ , Greer S , Casper M . Comparing methods of measuring geographic patterns in temporal trends: an application to county-level heart disease mortality in the United States, 1973 to 2010. Ann Epidemiol 2015;25(5):329–335.e3. 10.1186/1478-7954-11-8 25776848 PMC4397179

[10] Ben-Assuli O , Padman R , Bowman M , Leshno M , Shabtai I . On analyzing readmissions using a trajectory model: evidence from Israel. Stud Health Technol Inform 2015;216:1063. 26262362

[11] Hybels CF , Bennett JM , Landerman LR , Liang J , Plassman BL , Wu B . Trajectories of depressive symptoms and oral health outcomes in a community sample of older adults. Int J Geriatr Psychiatry 2016;31(1):83–91. 10.1002/gps.4292 25962827 PMC4641817

[12] Kwon S , Lee J , Carnethon MR . Developmental trajectories of physical activity and television viewing during adolescence among girls: National Growth and Health Cohort Study. BMC Public Health 2015;15(1):667. 10.1186/s12889-015-2043-4 26174016 PMC4502939

[13] Mostazir M , Jeffery A , Voss L , Wilkin T . Childhood obesity: evidence for distinct early and late environmental determinants a 12-year longitudinal cohort study (EarlyBird 62). Int J Obes 2015;39(7):1057–62. Erratum in: Int J Obes (Lond) 2016;40(2):380. 10.1038/ijo.2015.68 26857269

[14] National Center for Health Statistics. Compressed mortality file, 1999–2016 (machine readable data file and documentation, CD‑ROM Series 20, No. 2V) as compiled from data provided by the 57 vital statistics jurisdictions through the Vital Statistics Cooperative Program. Hyattsville (MD): National Center for Health Statistics; 2017.

[15] Jones B , Nagin D , Roeder K . A SAS procedure based on mixture models for estimating developmental trajectories. Sociol Methods Res 2001;29(3):374–93. 10.1177/0049124101029003005

[16] Jones B , Nagin D . Advances in group-based trajectory modeling and an SAS procedure for estimating them. Sociol Methods Res 2007;35(4):542–71. 10.1177/0049124106292364

[17] Nagin D . Analyzing developmental trajectories: semi-parametric, group-based approach. Psychol Methods 1999;4(2):139–57. 10.1037/1082-989X.4.2.139 11285809

[18] Nagin D . Group-based modeling of development. Boston (MA): Harvard University Press; 2005.

[19] Getis A . A geographic approach to identifying disease clusters. In: Janelle DG WB, Hansen K, editors. Worldminds: geographical perspectives on 100 problems: commemorating the 100th anniversary of the Association of American Geographers 1904–2004. Norwell (MA): Kluwer Academic Publishers; 2004.

[20] Zhou Y , Hallisey EJ , Freymann GR . Identifying perinatal risk factors for infant maltreatment: an ecological approach. Int J Health Geogr 2006;5(1):53. 10.1186/1476-072X-5-53 17144919 PMC1698478

[21] Singh GK , Siahpush M . Increasing inequalities in all-cause and cardiovascular mortality among US adults aged 25-64 years by area socioeconomic status, 1969-1998. Int J Epidemiol 2002;31(3):600–13. 10.1093/ije/31.3.600 12055162

[22] Wingate MS , Barfield WD , Smith RA , Petrini J . Perinatal disparities between American Indians and Alaska Natives and other US populations: comparative changes in fetal and first day mortality, 1995–2008. Matern Child Health J 2015;19(8):1802–12. 10.1007/s10995-015-1694-1 25663653

[23] Howard G , Peace F , Howard VJ . The contributions of selected diseases to disparities in death rates and years of life lost for racial/ethnic minorities in the United States, 1999–2010. Prev Chronic Dis 2014;11:E129. 10.5888/pcd11.140138 25078566 PMC4124043

[24] Hutchinson RN , Shin S . Systematic review of health disparities for cardiovascular diseases and associated factors among American Indian and Alaska Native populations. PLoS One 2014;9(1):e80973. 10.1371/journal.pone.0080973 24454685 PMC3893081

[25] Compiled from the census of 1860. (n.d.). Washington (DC): Library of Congress. https://www.loc.gov/resource/g3861e.cw0013200/?r=-0.195,-0.065,1.428,0.887,0 and https://www.loc.gov/resource/g3861e.cw0013200/?r=-0.195,-0.065,1.428,0.887,0. Accessed August 24, 2018.

[26] Isserman A . Appalachia then and now: update of “The Realities of Deprivation” reported to the president in 1964. J Appalach Stud 1997;3(1):43–69. http://www.arc.gov/assets/research_reports/socioeconomicreviewofappalachiathenandnow.pdf. Accessed March 21, 2019

[27] Leonard T , Hughes AE , Donegan C , Santillan A , Pruitt SL . Overlapping geographic clusters of food security and health: where do social determinants and health outcomes converge in the U.S? SSM Popul Health 2018;5:160–70. 10.1016/j.ssmph.2018.06.006 29998188 PMC6039352

[28] Franzini L , Ribble JC , Keddie AM . Understanding the Hispanic paradox. Ethn Dis 2001;11(3):496–518. 11572416

[29] Palloni A , Arias E . Paradox lost: explaining the Hispanic adult mortality advantage. Demography 2004;41(3):385–415. 10.1353/dem.2004.0024 15461007

